# Supplementary material for: Biological invasions facilitate zoonotic disease emergences
Source: Nat Commun. 2022 Apr 1;13:1762. doi: 10.1038/s41467-022-29378-2 (PMC8975888; doi:10.1038/s41467-022-29378-2)
Supplement: Supplementary file 1 — Supplementary Information [file 41467_2022_29378_MOESM1_ESM.pdf]

Supplementary Information file

Biological invasions facilitate zoonotic disease emergences

Zhang et al.

Supplementary Figures

Supplementary Fig. 1 Continental distribution of 10,473 zoonosis events from the year of 1348 to 2020 partitioned by (A) zoonotic animal host taxa and (B) pathogen taxa. AF: Africa, AS: Asia, EU: Europe, OA: Oceania, SA: South America, NA: North America.

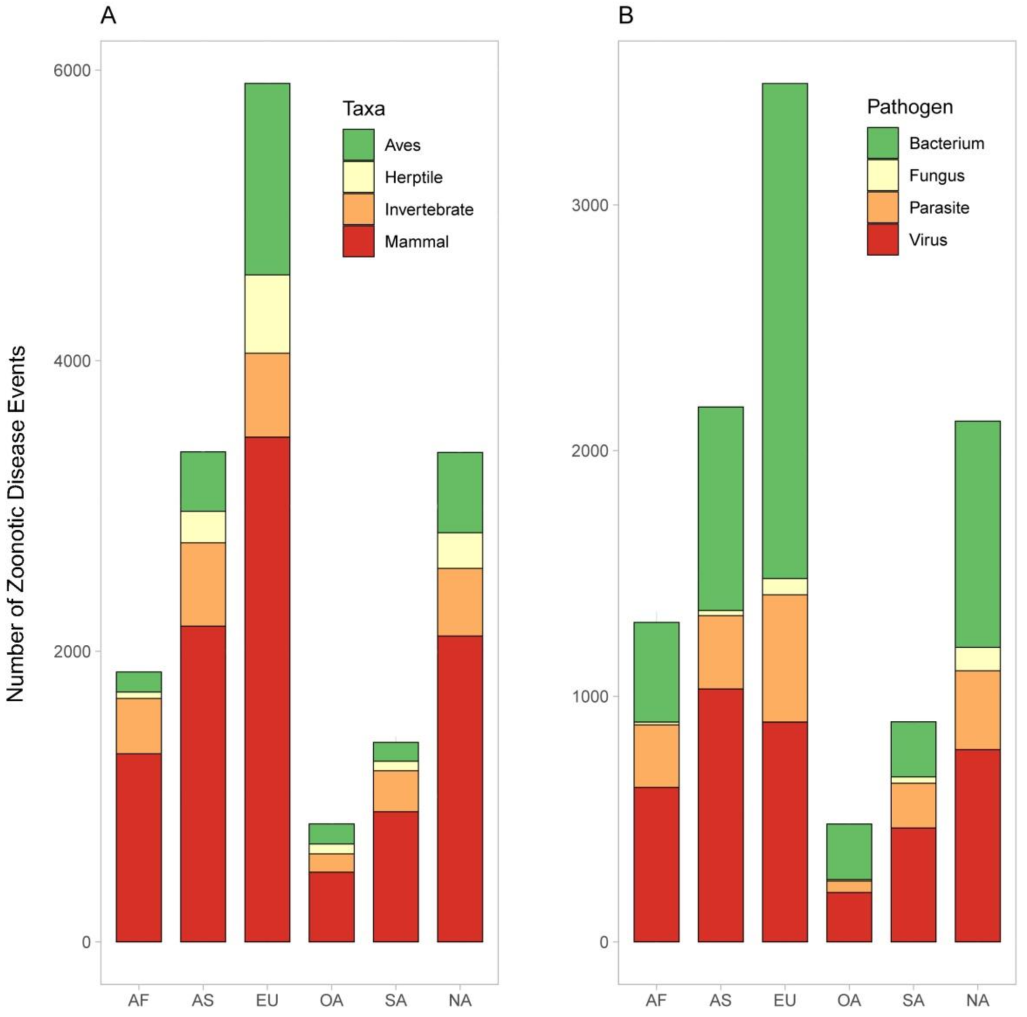

Supplementary Fig. 2 Global spatial patterns of 10,473 events of zoonotic diseases across 201 administrative areas based on (A) all reservoir zoonotic hosts or (B) mammalian, (C) avian, (D) herpetofaunal, or (E) invertebrate zoonotic hosts. The values are calculated by summing all reported zoonosis events caused by each order of the four taxa. Small circles represent islands or archipelagos. Animal silhouettes are from PhyloPic (http://phylopic.org/).

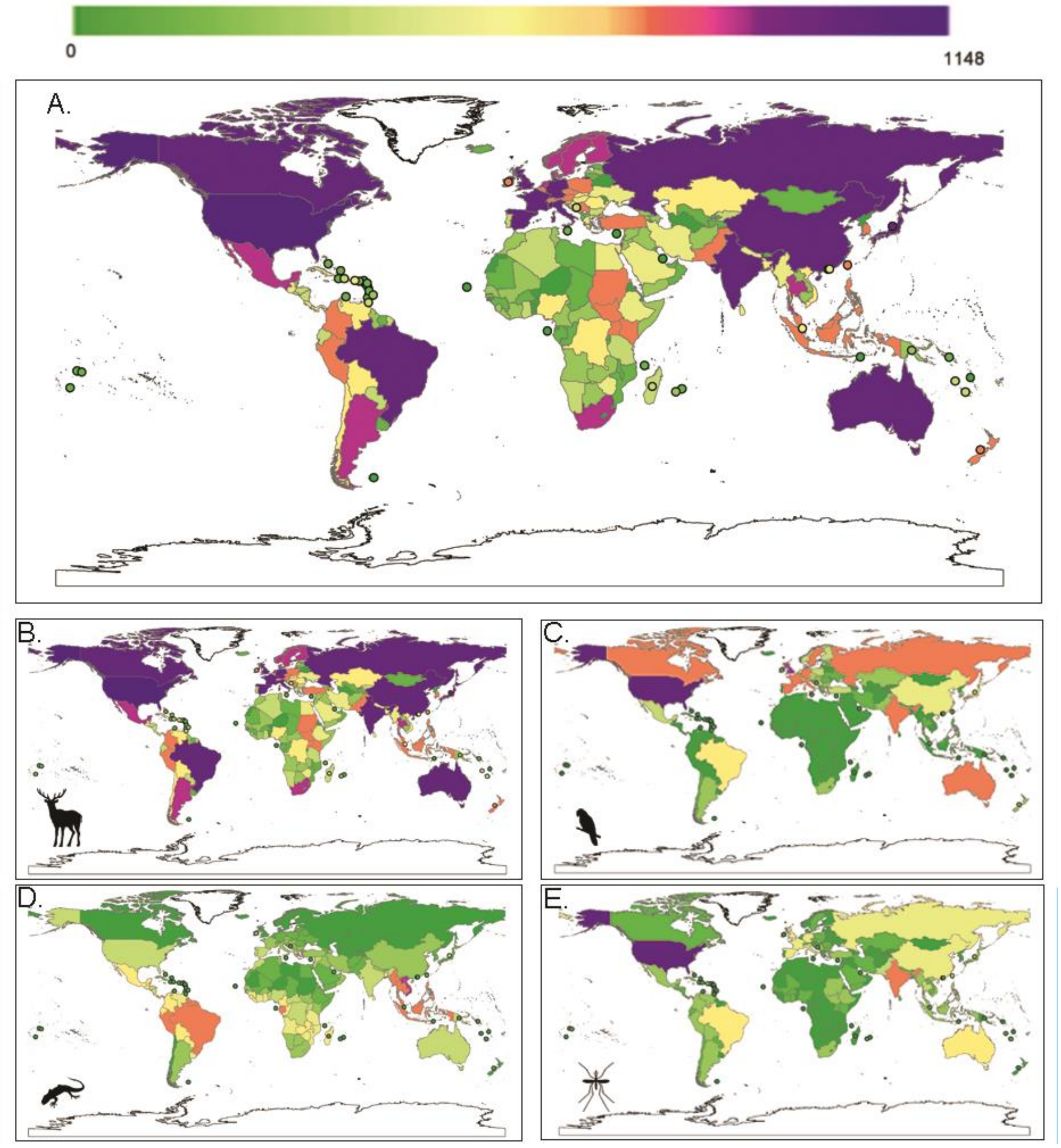

Supplementary Fig. 3 Proportion of deviance explained (A) and effect size of each predictor variable (B) in model averaging analyses based on GAMMs. Rows represent individual models and columns represent predictor variables with smoothing function *k* value = 6 and 8. Variables appear in all five most highly supported models in panel (A) with model-averaged 95% confidence intervals that do not overlap zero in panel (B) are shown in bold. The circle size in panel (A) represents the proportion of deviance explained by each predictor and the blank indicates that the predictors are not included in the model. The panel (B) represents mean effect size with 95% confidence intervals of different predictor variables explaining the number of zoonosis events worldwide (n = 10,473).

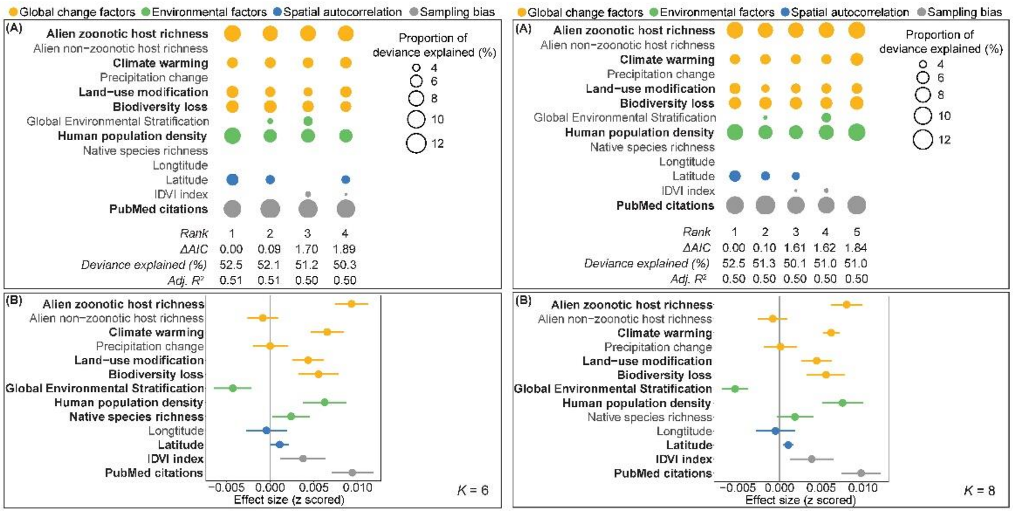

Supplementary Fig. 4 Scatter plots between alien zoonotic host introductions and the number of zoonosis events through time after controlling for the effect of non-zoonotic host effect. The line demonstrates the general tendency fitting with the mean and 95% confidence interval based on generalized additive model (GAM).

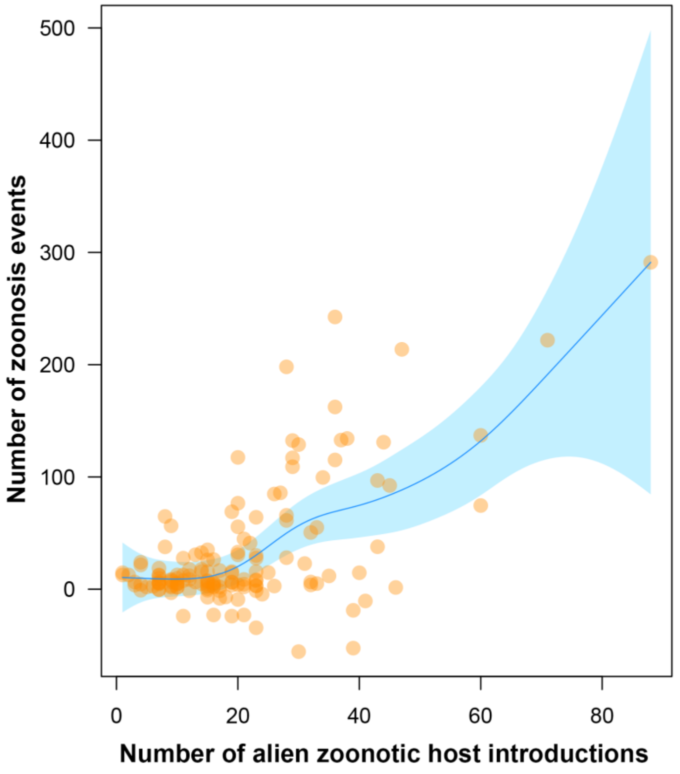

Supplementary Fig. 5 Breakpoint regression models showing the number of zoonotic disease events (A), the number of alien zoonotic host introductions (B), and the number of alien non-zoonotic host introductions (C) during the years of 1800-2000. The performance of each model in each section is assessed by using the Akaike information criterion based on a small sample size (AICc). Abbreviations: N, the number of zoonotic disease events (cumulative), or the alien zoonotic or non-zoonotic species introductions; Y, year; c, intercept; zi, slope; T, breakpoint year. The best model that predicts the optimal time of the increase of human avian-hosted zoonosis events and the number of alien zoonotic or zoonotic avian introductions are marked in bold. The green dashed line indicates the optimal breakpoint year. The number of zoonosis events and alien zoonotic or non-zoonotic host introductions are calculated by combining the mammalian-, avian- and invertebrate cases together.

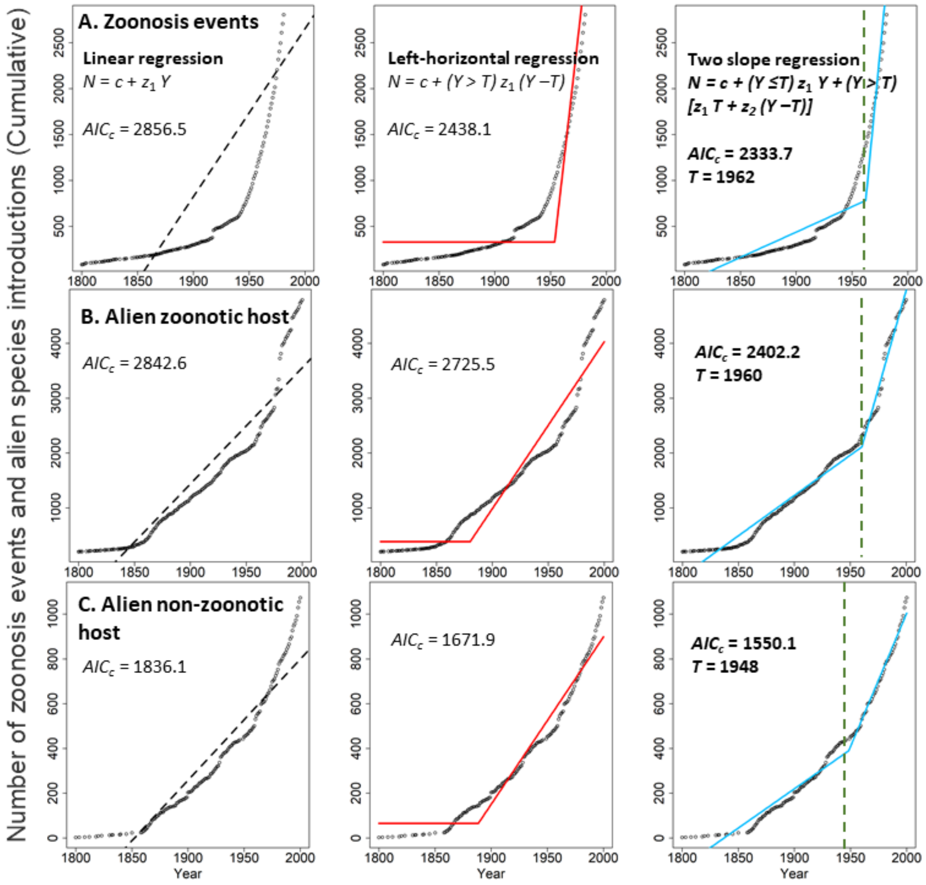

Supplementary Fig. 6 Correlations among all predictor variables used in the GAMM analyses based on Pearson rank correlation analyses.

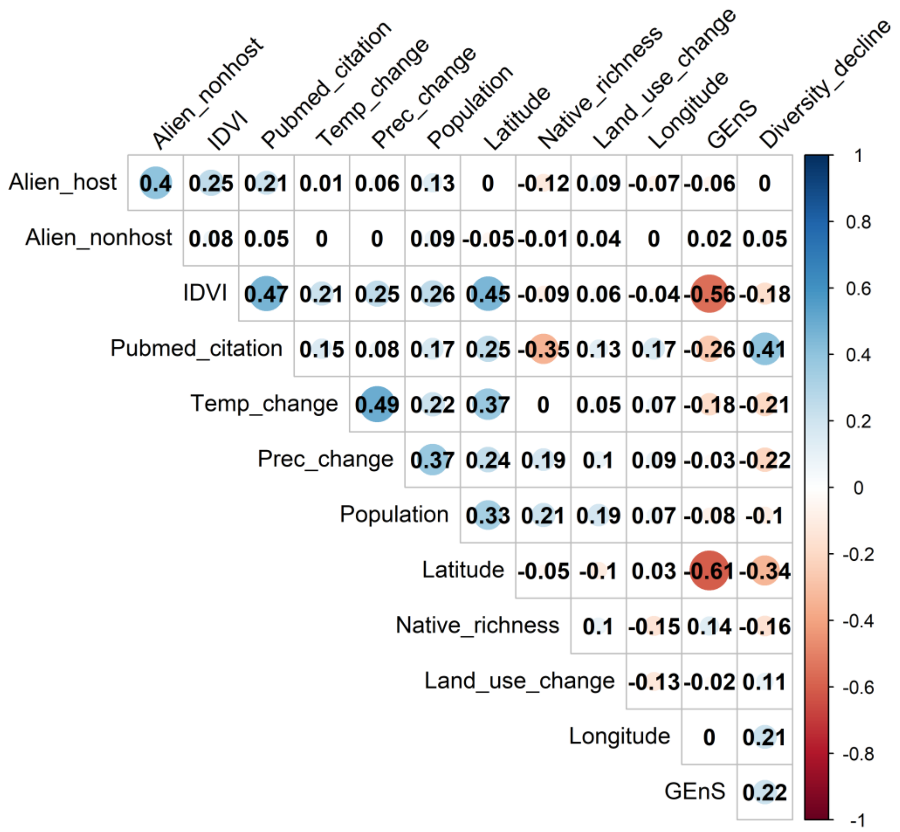

Supplementary Notes

R codes used for model averaging analyses based on GAMM to quantify the relationship between different predictor variables and the number of zoonotic disease events (A), and for breakpoint regression analyses to explore the temporal relationship of alien zoonotic (and non-zoonotic) host introductions and zoonotic diseases over years (B).

(A)

<u>Abbreviations</u>

zgamm.csv: file name. *resdv*: residuals of the fitted regression correlating the density of zoonosis events and the density of all disease events, *alienhost*: density of alien zoonotic species richness, *nonhost*: density of alien non-zoonotic species richness, *natdensity*: density of native species richness, *ges*: global environmental stratification, population: human population density, *landuse*: the degree of land-use type changed, *warming*: the slope of the temperature for the years 1901 to 2009, *prech*: the slope of the precipitation for the years 1901 to 2009, *biodec*: biodiversity loss, *idvi*: Infectious Disease Vulnerability Index, pub: PubMed citations, *lat/lon*: latitude and longitude of the geographic centroid of administrative units; *continent*: continental identity as a random intercept, *pathogen*: pathogen identity as a random intercept, *order*: host order as a random intercept. *k*: thin-plate spline smooths for each predictor variable.

```
library(gamm4)
library(mgcv)
library(visreg)
library(MuMIn)
library(dplyr)

a = read.csv(' zgamm.csv')
a$continent<-factor(a$continent)
a$pathogen<-factor(a$pathogen)
a$order<-factor(a$order)
levels(a$order)
a$order <- relevel(a$order, ref="control")
Terms <- list(
   nonhostsv = c(NA, "s(nonhostsv,k=10)"),
   natdensity = c(NA, "s(natdensitysv,k=10)"),
   ges = c(NA, "s(geslogsv,k=10)"),
   population = c(NA, "s(populationsv,k=10)"),
   alienhost = c(NA, "s(alienhostsv,k=10)"),
   landuse = c(NA, "s(landusesv,k=10)"),
   warming = c(NA, "s(warmingsv,k=10)"),
   biodec = c(NA, "s(biodeclogsv,k=10)"),
   idvi = c(NA, "s(idvisv,k=10)"),
   pub = c(NA, "s(publogsv,k=10)"),
   prech = c(NA, "prechsv"),
```

```
   lat = c(NA, "s(latsv,k=10)"),
   log = c(NA, "s(logsv,k=10)"),
   continent = c("s(continent, bs = 're')"),
   pathogen = c("s(pathogen, bs = 're')"),
   order = c("s(order, bs = 're')"))

## All possible combinations of these terms:
CompetingFullModels <- expand.grid(Terms)

## Build formulas
CompetingFullModels <- apply(CompetingFullModels, 1, function(row)
paste(na.omit(row), collapse = ' + ')) %>%
   data.frame(Formula = ., stringsAsFactors = F) %>%
   mutate(Formula = ifelse(nchar(Formula) == 0, '1', Formula),
             Formula = paste('resdv ~', Formula))

## Model fit
CompetingFits <- CompetingFullModels %>%
   group_by(Formula) %>%
   do(ModelFit = try(gam(as.formula(.$Formula),
                                   family = 'gaussian',
                                   data = a,
                                   select = FALSE,
                                   method = 'REML')))
gc()
removeRows <- lapply(CompetingFits$ModelFit, function(x) 'try-error' %in%
class(x)) %>%
   unlist()
FailedFormulas <- CompetingFits[removeRows, ]
stopifnot(nrow(FailedFormulas) == 0)

CompetingFits <- CompetingFits[!removeRows, ]


## Add AIC:
RankedModels <- CompetingFits %>%
   mutate(AIC = AIC(ModelFit),
             DevianceExplained = summary(ModelFit)$dev.expl,
             Adj_R_square = summary(ModelFit)$r.sq) %>%
   ungroup() %>%
   arrange(AIC) %>%
   mutate(DeltaAIC = AIC - AIC[1])

## Save fitted models' results
```

```
RankedModels %>%
  select(-ModelFit) %>%
  write.csv('RankedModels.csv', row.names = FALSE)
```

(B)

Abbreviations

zidtime.*csv*: file name. *ceid*: number of zoonosis event; this code is used to identify the breakpoint reflecting the year in which there was a rapid increase in the number zoonotic diseases, which can also be used to identify the breakpoint of the increase of zoonotic or non-zoonotic host species with year.

```
library(segmented)
a=read.csv("zidtime.csv", header=T)
fit.lm <- lm(ceid~year, data = a)
summary(fit.lm)
AICc(fit.lm)
year1 <- a$year
fit.lm.0<- lm(ceid~1,data = a)
fit.seg.0<- segmented(fit.lm.0,seg.Z = ~year1)
summary(fit.seg.0)
AICc(fit.seg.0)
fit.seg<- segmented(fit.lm,~year1)
summary(fit.seg)
AICc(fit.seg.0)
```
