# Supplementary material for: Biological invasions facilitate zoonotic disease emergences
Source: Nat Commun. 2022 Apr 1;13:1762. doi: 10.1038/s41467-022-29378-2 (PMC8975888; doi:10.1038/s41467-022-29378-2)
Supplement: Supplementary file 3 — Description of Additional Supplementary Information [file 41467_2022_29378_MOESM3_ESM.docx]

**Inventory of Supporting Information**

Supplementary Fig. 1 Continental distribution of 10,473 zoonosis events from the year of 1348 to 2020 partitioned by (A) zoonotic animal host taxa and (B) pathogen taxa. AF: Africa, AS: Asia, EU: Europe, OA: Oceania, SA: South America, NA: North America.

Supplementary Fig. 2 Global spatial patterns of 10,473 events of human zoonotic EIDs across 201 administrative areas based on (A) all reservoir zoonotic hosts or (B) mammalian, (C) avian, (D) herpetofaunal, or (E) invertebrate zoonotic hosts. The values were calculated by summing all reported human EID events caused by each order of the four taxa. Small circles represent islands or archipelagos. Animal silhouettes from PhyloPic.

Supplementary Fig. 3 Proportion of deviance explained (A) and effect size of each predictor variable (B) in model averaging analyses based on GAMMs. Rows represent individual models and columns represent predictor variables with smoothing function k value = 6 and 8. Variables appear in all five most highly supported models in panel (A) with model-averaged 95% confidence intervals that do not overlap zero in panel (B) are shown in bold. The circle size in panel (A) represents the proportion of deviance explained by each predictor and the blank indicated that the predictors are not included in the model. The panel (B) represents mean effect size with 95% confidence intervals of different predictor variables explaining the number of zoonosis events across pathogens, host orders and continents (n = 10,473).

Supplementary Fig. 4 Scatter plots between alien zoonotic host introductions and the number of zoonosis events through time after controlling for the effect of non-zoonotic host effect. The line demonstrates the general tendency fitting with the mean and 95% confidence interval based on generalized additive model (GAM).

Supplementary Fig. 5 Breakpoint regression models showing the number of zoonotic disease events (A), the number of alien zoonotic host introductions (B), and the number of alien non-zoonotic host introductions (C) during the years of 1800-2000. The performance of each model in each section was assessed by using the Akaike information criterion based on a small sample size (AICc). Abbreviations: N, the number of zoonotic disease events (cumulative), or the alien zoonotic or non-zoonotic species introductions; Y, year; c, intercept; zi, slope; T, breakpoint year. The best model that predicted the optimal time of the increase of human avian-hosted EID events and the number of alien zoonotic or zoonotic avian introductions are marked in bold. The green dashed line indicated the optimal breakpoint year. The number of EID events and alien zoonotic or non-zoonotic host introductions were calculated by combining the mammalian-, avian- and invertebrate cases together.

Supplementary Fig. 6 Correlations among all predictor variables used in the GAMM analyses based on Pearson rank correlation analyses.

Supplementary Data 1. Number of zoonosis events in the 201 administrative areas across host taxonomic groups recorded by the GIDEION database.

Supplementary Data 2. Literature and database used to identify the evidence of the established alien animals as zoonotic hosts of the 161 zoonoses across different pathogens in the GIDEON database. Those established alien species that don't carry zoonoses in the GIDEON database are not shown in this table.

Supplementary Data 3. Number of zoonoses reported by the GIDEON database across alien zoonotic host groups.

Supplementary Data 4. The predictor variables used to explore the important factors associated with zoonosis emergences across alien host groups at the global scale.

Supplementary Notes. R code used for model averaging analyses based on GAMM to quantify the relationship between different predictor variables and the number of zoonotic disease events (A), and for breakpoint regression analyses to explore the temporal relationship of alien zoonotic (and non-zoonotic) host introductions and zoonotic diseases over years (B).
