# Supplementary material for: Biological invasions facilitate zoonotic disease emergences
Source: Nat Commun. 2022 Apr 1;13:1762. doi: 10.1038/s41467-022-29378-2 (PMC8975888; doi:10.1038/s41467-022-29378-2)
Supplement: Supplementary file 8 — Reporting Summary [file 41467_2022_29378_MOESM8_ESM.pdf]

nature portfolio

Corresponding author(s): Xuan Liu

Last updated by author(s): Feb 1, 2022

Reporting Summary

Nature Portfolio wishes to improve the reproducibility of the work that we publish. This form provides structure for consistency and transparency in reporting. For further information on Nature Portfolio policies, see our Editorial Policies and the Editorial Policy Checklist.

Statistics

For all statistical analyses, confirm that the following items are present in the figure legend, table legend, main text, or Methods section.

n/a	Confirmed	
☐	☒	The exact sample size (*n*) for each experimental group/condition, given as a discrete number and unit of measurement
☒	☐	A statement on whether measurements were taken from distinct samples or whether the same sample was measured repeatedly
☐	☒	The statistical test(s) used AND whether they are one- or two-sided<br>*Only common tests should be described solely by name; describe more complex techniques in the Methods section.*
☐	☒	A description of all covariates tested
☐	☒	A description of any assumptions or corrections, such as tests of normality and adjustment for multiple comparisons
☐	☒	A full description of the statistical parameters including central tendency (e.g. means) or other basic estimates (e.g. regression coefficient) AND variation (e.g. standard deviation) or associated estimates of uncertainty (e.g. confidence intervals)
☐	☒	For null hypothesis testing, the test statistic (e.g. *F*, *t*, *r*) with confidence intervals, effect sizes, degrees of freedom and *P* value noted<br>*Give P values as exact values whenever suitable.*
☒	☐	For Bayesian analysis, information on the choice of priors and Markov chain Monte Carlo settings
☒	☐	For hierarchical and complex designs, identification of the appropriate level for tests and full reporting of outcomes
☐	☒	Estimates of effect sizes (e.g. Cohen's *d*, Pearson's *r*), indicating how they were calculated

Our web collection on statistics for biologists contains articles on many of the points above.

Software and code

Policy information about availability of computer code

Data collection	A total of 161 known zoonotic EIDs were obtained from the GIDEON database. The exact source information used to identify whether the established alien animals are zoonotic hosts or non-zoonotic hosts of the 161 zoonoses were provided in Supplementary Data 2. The climatic variables were collected from the global environmental stratification (GEnS) database and University of East Anglia Climate Research Unit (CRU) database. The human population density data was collected from World Bank Open Data. The richness of native amphibians, birds, and mammals was derived from the Biodiversity Mapping website. The map of reptile diversity is based on an updated database of the global spatial distribution of reptiles (Roll et al. 2017; Nature Ecology & Evolution; https://doi.org/10.1038/s41559-017-0332-2). Data on 262 established alien herpetofauna were compiled from multiple publications, including Kraus (2009)'s compendium and Capinha et al. (2017)'s updates. Data on 337 established alien birds were collected from the Global Avian Invasions Atlas. Data on 119 established alien mammals were obtained from the Introduced Mammals of the World database and other reference updates. Data on 77 terrestrial alien invertebrates with available native and invaded range information were obtained from the Global Invasive Species Database (GISD). IDVI index of each country was from the reference of Moore et al. 2017 (https://pubmed.ncbi.nlm.nih.gov/28845357/). PubMed citations per disease for each jurisdiction were calculated using a Python-based PubCrawler tool (Allen et al. 2017; Nature Communications; https://doi.org/10.1038/s41467-017-00923-8). The global land-use data was downloaded from the Anthromes v2 Dataset.
Data analysis	All the main data analyses were performed using R version 4.0.3. GAMM and model averaging analyses were conducted in the gamm4 (version 0.2-6), mgcv (version 1.8-38), visreg (version 2.7.0), dplyr (version 1.0.0), and MuMIn (version 1.43.17) packages. Breakpoint regression analyses were conducted using the package segmented (version 1.4-0). All R codes used for these analyses were provided in Supplementary Notes.

For manuscripts utilizing custom algorithms or software that are central to the research but not yet described in published literature, software must be made available to editors and reviewers. We strongly encourage code deposition in a community repository (e.g. GitHub). See the Nature Portfolio guidelines for submitting code & software for further information.

Data

Policy information about availability of data

All manuscripts must include a data availability statement. This statement should provide the following information, where applicable:

- Accession codes, unique identifiers, or web links for publicly available datasets
- A description of any restrictions on data availability
- For clinical datasets or third party data, please ensure that the statement adheres to our policy

We used existing datasets for the analyses of the present study. The exact list of known zoonotic pathogens and zoonoses at each administrative region is from the GIDEON database (www.gideononline.com). The administrative designations used in our analyses were based on the Global Administrative Areas (GADM) database (www.gadm.org). The alien host-pathogen association data were based on Gibb's dataset (CLOVER_Associations_Initial.csv, https://dx.doi.org/10.5281/zenodo.4435128), and other literatures in Supplementary Data 2. The Infectious Disease Vulnerability Index (IDVI) was from Moore et al. (2017) (https://pubmed.ncbi.nlm.nih.gov/28845357/). We used global environmental stratification (GEnS, https://www.geoportal.org/) as a composite bioclimatic variable generated by stratifying the Earth's surface into zones with similar climates. For the temperature and precipitation change variable, we extracted historical monthly mean temperature and precipitation data recorded between 1901 and 2009 from the University of East Anglia Climate Research Unit (CRU, https://catalogue.ceda.ac.uk/uuid/3f8944800cc48e1cbc29a5ee12d8542d), and global land-use data from the Anthromes v2 Dataset (Anthropogenic Biomes version 2, https://ecotope.org/anthromes/v2/). Human population size data and the land area of each jurisdiction were collected from World Bank Open Data from 2011 to 2020 (available at https://data.worldbank.org/indicator/SP.POP. TOTL). Data on the richness of native amphibians, birds, and mammals were derived from the Biodiversity Mapping website (https://biodiversitymapping.org/wordpress/index.php/home/) derived from Jenkins et al. (2013) (https://doi.org/10.1073/pnas.1302251110) and Pimm et al. (2014) (https://www.science.org/doi/10.1126/science.1246752). The latest map for the reptile species was from Roll et al. (2017) (https://doi.org/10.5061/dryad.83s7k). The list of threatened species evaluated as NT, EN and VU used for calculating the loss of native biodiversity was extracted from the IUCN Red List (http://www.iucnredlist.org). The established alien species list and the introduction time information used for the temporal analyses are based on Kraus's (2009) compendium (https://link.springer.com/chapter/10.1007/978-1-4020-8946-6_6) and Capinha et al. (2017) (http://dx.doi.org/10.1111/ddi.12617) for reptiles and amphibians, the Global Avian Invasions Atlas (GAVIA) (http://dx.doi.org/10.6084/m9.figshare.4234850) for established alien birds, Long's (2003) book (https://ebooks.publish.csiro.au/content/introduced-mammals-world) and Capellini et al. (2015) (http://dx.doi.org/10.1111/ele.12493) for established alien mammals, and the Global Invasive Species Database (GISD, http://www.iucngisd.org/gisd/) for established alien vertebrates. All data used to conduct the model analyses were provided in Supplementary Data 4.

Field-specific reporting

Please select the one below that is the best fit for your research. If you are not sure, read the appropriate sections before making your selection.

☐ Life sciences ☐ Behavioural & social sciences ☒ Ecological, evolutionary & environmental sciences

For a reference copy of the document with all sections, see nature.com/documents/nr-reporting-summary-flat.pdf

Ecological, evolutionary & environmental sciences study design

All studies must disclose on these points even when the disclosure is negative.

Study description

Alien species invasions have not only caused devasting impacts on economies and biodiversity, but also have been regarded as one great threat to global public health by transmitting novel and re-emerging human diseases, yet a global synthesis of the role of established alien zoonotic hosts on human emerging infectious diseases (EIDs) across a broad range of taxonomic groups is lacking. Here, we isolate and quantify the effect of biological invasions on human zoonotic disease emergences after accounting for other co-factors and propagule pressure worldwide.

Research sample

We used existing datasets for the analyses of the present study. The exact list of known zoonotic pathogens and zoonoses at each administrative region is from the GIDEON database (www.gideononline.com). The administrative designations used in our analyses were based on the Global Administrative Areas (GADM) database (www.gadm.org). The alien host-pathogen association data were based on Gibb's dataset (CLOVER_Associations_Initial.csv, https://dx.doi.org/10.5281/zenodo.4435128), and other literatures in Supplementary Data 2. The Infectious Disease Vulnerability Index (IDVI) was from Moore et al. (2017) (https://pubmed.ncbi.nlm.nih.gov/28845357/). We used global environmental stratification (GEnS, https://www.geoportal.org/) as a composite bioclimatic variable generated by stratifying the Earth's surface into zones with similar climates. For the temperature and precipitation change variable, we extracted historical monthly mean temperature and precipitation data recorded between 1901 and 2009 from the University of East Anglia Climate Research Unit (CRU, https://catalogue.ceda.ac.uk/uuid/3f8944800cc48e1cbc29a5ee12d8542d), and global land-use data from the Anthromes v2 Dataset (Anthropogenic Biomes version 2, https://ecotope.org/anthromes/v2/). Human population size data and the land area of each jurisdiction were collected from World Bank Open Data from 2011 to 2020 (available at https://data.worldbank.org/indicator/SP.POP. TOTL). Data on the richness of native amphibians, birds, and mammals were derived from the Biodiversity Mapping website (https://biodiversitymapping.org/wordpress/index.php/home/) derived from Jenkins et al. (2013) (https://doi.org/10.1073/pnas.1302251110) and Pimm et al. (2014) (https://www.science.org/doi/10.1126/science.1246752). The latest map for the reptile species was from Roll et al. (2017) (https://doi.org/10.5061/dryad.83s7k). The list of threatened species evaluated as NT, EN and VU used for calculating the loss of native biodiversity was extracted from the IUCN Red List (http://www.iucnredlist.org).
The established alien species list and the introduction time information used for the temporal analyses are based on Kraus's (2009) compendium (https://link.springer.com/chapter/10.1007/978-1-4020-8946-6_6) and Capinha et al. (2017) (http://dx.doi.org/10.1111/ddi.12617) for reptiles and amphibians, the Global Avian Invasions Atlas (GAVIA) (http://dx.doi.org/10.6084/m9.figshare.4234850) for established alien birds, Long's (2003) book (https://ebooks.publish.csiro.au/content/introduced-mammals-world) and Capellini et al. (2015) (http://dx.doi.org/10.1111/ele.12493) for established alien mammals, and the Global Invasive Species Database (GISD, http://www.iucngisd.org/gisd/) for established alien vertebrates.

Sampling strategy	We identified the zoonotic emerging infectious disease used in the present study and the alien zoonotic host richness of each country or region for each taxon based on careful collections from various widely used databases on zoonotic diseases such as GIDEON and different global alien animal establishments for each taxon, and an intensive review of published references which are both provided in supporting data. As this is not an experimental study and our analyses are conducted at the global scale, we used all available data from those global datasets above and there is no need to predetermine sample sizes.
Data collection	The datasets used above were downloaded and collected by Lin Zhang, Ruina Cui, Yusi Xin, Xuan Liu, Lixia Han, Jing Liang, Xuyu Wang, Xiaona Yang and Zhengjun Wu.
Timing and spatial scale	We collected the disease data from the GIDEON database and the administrative designations information from the Global Administrative Areas (GADM) database on November, 2020. We obtained the human population density data from World Bank Open Data from 2011 to 2020 on November 18, 2020. Data on the richness of native amphibians, birds, and mammals were derived from the Biodiversity Mapping website on August 19, 2020. Data on 77 terrestrial alien invertebrates were collected from the Global Invasive Species Database (GISD) on July 1, 2020. We obtained the historical climates from CRU on November 30, 2020. We downloaded global land-use data from the Anthromes v2 Dataset on October 15, 2020. The list of threatened species was extracted from the IUCN Red List on May 10th, 2021. All datasets downloaded and used in the study are at the global scale.
Data exclusions	As the aim of our present study is to test the role of established alien animal species in the spread of zoonotic diseases, we excluded diseases with human-specific hosts that do not need animals to persist or be transmitted.
Reproducibility	As this is not an experimental study but conducted analyses using existing databases, we did not test for reproducibility of experiments. We have provided the supplementary data and R codes to reproduce the main findings of the present study.
Randomization	As this is not an experimental study but analyzed existing datasets, we did not have to allocate samples randomly to groups.
Blinding	Blinding was not relevant with our study because all data used in the present study was collected from public databases and published references, which have been provided in the supporting data.

Did the study involve field work? ☐ Yes ☒ No

Reporting for specific materials, systems and methods

We require information from authors about some types of materials, experimental systems and methods used in many studies. Here, indicate whether each material, system or method listed is relevant to your study. If you are not sure if a list item applies to your research, read the appropriate section before selecting a response.

Materials & experimental systems

n/a	Involved in the study
☒	☐ Antibodies
☒	☐ Eukaryotic cell lines
☒	☐ Palaeontology and archaeology
☒	☐ Animals and other organisms
☒	☐ Human research participants
☒	☐ Clinical data
☒	☐ Dual use research of concern

Methods

n/a	Involved in the study
☒	☐ ChIP-seq
☒	☐ Flow cytometry
☒	☐ MRI-based neuroimaging
